# Supplementary material for: ‘We don’t see because we don’t ask’: Qualitative exploration of service users’ and health professionals’ views regarding a psychosocial intervention targeting pregnant women experiencing domestic and family violence
Source: PLoS One. 2020 Mar 9;15(3):e0230069. doi: 10.1371/journal.pone.0230069 (PMC7062263; doi:10.1371/journal.pone.0230069)
Supplement: S1 COREQ checklist — (DOCX) [file pone.0230069.s001.DOCX]

**Consolidated criteria for reporting qualitative studies (COREQ): 32-item checklist**

Developed from:

Tong A, Sainsbury P, Craig J. Consolidated criteria for reporting qualitative research (COREQ): a 32-item checklist for interviews and focus groups. *International Journal for Quality in Health Care*. 2007. Volume 19, Number 6: pp. 349 – 357

**YOU MUST PROVIDE A RESPONSE FOR ALL ITEMS. ENTER N/A IF NOT APPLICABLE**

| **No. Item** | **Guide questions/description** |  | **Reported on Page #** |
| --- | --- | --- | --- |
| **Domain 1: Research team and reﬂexivity** |  |  |  |
| *Personal Characteristics* |  |  |  |
| 1. Inter viewer/facilitator | Which author/s conducted the inter view or focus group? | DS and RP | Materials and Methods (Page 8) |
| 2. Credentials | What were the researcher’s credentials? E.g. PhD, MD | RN, MSc Nursing, PhD, MPH, MD (Obstetrics and Gynaecology) | Title page |
| 3. Occupation | What was their occupation at the time of the study? | DS and RP are registered nurses and have experience in qualitative research. DS is currently a PhD Candidate in School of Nursing and RS is an Assistant Professor of Psychiatric Nursing. | Materials and Methods (Page 8) and Title page |
| 4. Gender | Was the researcher male or female? | Female except PR, who is a male | N/A |
| 5. Experience and training | What experience or training did the researcher have? | AS, KB, DA have PhD degree and have supervised several PhD students in conducting both quantitative and qualitative research. DS is a PhD candidate. RP is an Assistant Professor in psychiatric nursing. AS, KB, DA, RP and DS are registered nurses and PR is an obstetrician and gynecologist. DS has experience in conducting research projects employing qualitative methodologies. Both DS and RP have studied about the qualitative research in detail during their masters and are experienced in conducting qualitative interviews. | Title page |
| *Relationship with participants* |  |  | N/A |
| 6. Relationship established | Was a relationship established prior to study commencement? | None of the participants had an established relationship with any of the authors prior to study commencement. | N/A |
| 7. Participant knowledge of the interviewer | What did the participants know about the researcher? e.g. personal goals, reasons for doing the research | Participants were informed that the researchers were interested in collecting their feedback regarding the psychosocial intervention. They were informed that it was a part of a PhD project of DS. They were provided with the participants’ information sheet outlining purpose and procedures of the study at the time of recruitment prior to giving their written informed consent to be involved. | N/A |
| 8. Interviewer characteristics | What characteristics were reported about the inter viewer/facilitator? e.g. Bias, assumptions, reasons and interests in the research topic | A brief introduction about the interviewers including organizational affiliations, their names and positions were provided to the participants before starting the interview. No any interviewer-related biases were identified. | N/A |
| **Domain 2: study design** |  |  |  |
| *Theoretical framework* |  |  |  |
| 9. Methodological orientation and Theory | What methodological orientation was stated to underpin the study? e.g. grounded theory, discourse analysis, ethnography, phenomenology, content analysis | Open and axial coding with thematic content analysis. | Materials and Methods (Page 9) |
| *Participant selection* |  |  |  |
| 10. Sampling | How were participants selected? e.g. purposive, convenience, consecutive, snowball | All participants from the intervention group were selected and health care providers were selected purposively. | Materials and Methods (Page 8) |
| 11. Method of approach | How were participants approached? e.g. face-to-face, telephone, mail, email | Face-to-face interviews were conducted. For some women, telephone interview was conducted. | Materials and Methods (Page 8) |
| 12. Sample size | How many participants were in the study? | All women from the intervention group and 7 health care providers were interviewed. | Results (Page 11) |
| 13. Non-participation | How many people refused to participate or dropped out? Reasons? | 7 participants at the first follow-up and 19 at the second follow-up were lost to follow-up; thus, it was not possible to interview them. There were no participants who subsequently refused to participate, withdrew consent or dropped out. | Discussion (Page 28) |
| *Setting* |  |  |  |
| 14. Setting of data collection | Where was the data collected? e.g. home, clinic, workplace | All interviews were conducted in the hospital (at antenatal clinic for women and at their offices for health care providers). | Materials and Methods (Page 8) |
| 15. Presence of non-participants | Was anyone else present besides the participants and researchers? | No | N/A |
| 16. Description of sample | What are the important characteristics of the sample? e.g. demographic data, date | Pregnant women (24-34 weeks of gestation) having a history of DFV were recruited in the study. A total of seven HCPs (3 nurses, 2 obstetricians/gynaecologist, 1 health manager, and 1 Midwifery professor) were interviewed. | Results (Page 11) |
| *Data collection* |  |  |  |
| 17. Interview guide | Were questions, prompts, guides provided by the authors? Was it pilot tested? | Interview guide was prepared and it was rigorously discussed among the authors. Probes were used to facilitate discussions. | Materials and Methods (Page 8) |
| 18. Repeat interviews | Were repeat inter views carried out? If yes, how many? | No | N/A |
| 19. Audio/visual recording | Did the research use audio or visual recording to collect the data? | Interviews were audio recorded. | Materials and Methods (Page 8) |
| 20. Field notes | Were ﬁeld notes made during and/or after the inter view or focus group? | Hand written notes were kept at the time of discussion. | Materials and Methods (Page 8) |
| 21. Duration | What was the duration of the inter views or focus group? | Interviews with women lasted for about 15-20 minutes and with health care providers, each interview was of an average 30-45 minutes. | Materials and Methods (Page 8) |
| 22. Data saturation | Was data saturation discussed? | No. | N/A |
| 23. Transcripts returned | Were transcripts returned to participants for comment and/or correction? | No | N/A |
| **Domain 3: analysis and ﬁndings** |  |  |  |
| *Data analysis* |  |  |  |
| 24. Number of data coders | How many data coders coded the data? | Two authors (DS and RP) coded the data. | Materials and Methods (Page 9) |
| 25. Description of the coding tree | Did authors provide a description of the coding tree? | Yes, the authors generated a coding system in MS Excel with codes, categories and themes generated. | N/A |
| 26. Derivation of themes | Were themes identiﬁed in advance or derived from the data? | Themes were derived from the data. However, the main goal of the study was to identify strengths and weaknesses of the piloted intervention, which informed the structure of the themes. | Materials and Methods (Page 9) |
| 27. Software | What software, if applicable, was used to manage the data? | Microsoft Word and Microsoft Excel | Materials and Methods (Page 9) |
| 28. Participant checking | Did participants provide feedback on the ﬁndings? | No | N/A |
| *Reporting* |  |  |  |
| 29. Quotations presented | Were participant quotations presented to illustrate the themes/ﬁndings? Was each quotation identiﬁed? e.g. participant number | Yes, quotations were presented to illustrate the themes/findings, and each quotation was identified with an anonymous participant identifier. | Results (Page 12-24) |
| 30. Data and ﬁndings consistent | Was there consistency between the data presented and the ﬁndings? | Yes, there was consistency between the data presented and the findings. | Discussion (Page 24-28) |
| 31. Clarity of major themes | Were major themes clearly presented in the ﬁndings? | Yes, major themes were clearly presented in the Results section using specific sections regarding each theme. | Results (Page 11-24), Fig 1 |
| 32. Clarity of minor themes | Is there a description of diverse cases or discussion of minor themes? | No | N/A |

**Once you have completed this checklist, please save a copy and upload it as part of your submission. When requested to do so as part of the upload process, please select the file type: *Checklist*. You will NOT be able to proceed with submission unless the checklist has been uploaded. Please DO NOT** **include this checklist as part of the main manuscript document. It must be uploaded as a separate file.**
