# Supplementary material for: ‘We don’t see because we don’t ask’: Qualitative exploration of service users’ and health professionals’ views regarding a psychosocial intervention targeting pregnant women experiencing domestic and family violence
Source: PLoS One. 2020 Mar 9;15(3):e0230069. doi: 10.1371/journal.pone.0230069 (PMC7062263; doi:10.1371/journal.pone.0230069)
Supplement: S1 Interview schedule — (DOCX) [file pone.0230069.s002.docx]

**Interview Schedule for Intervention participants**

As you have participated in the intervention, we would like to seek your feedback regarding the intervention.

1. How do you find the intervention program provided to you at the hospital?
2. Which component of the intervention was most useful and why?
   1. Counselling session
   2. Information booklet
   3. Telephone support
3. What do you think were the strengths of this intervention?
4. In your opinion, what were the weaknesses of this intervention?
5. Do you have any recommendations/suggestions related to the intervention? If yes, can you please elaborate?
6. At the end, do you have anything to share with us regarding the intervention? If yes, can you please describe it briefly?

**Interview schedule for health care providers**

1. In your opinion, how prevalent is the domestic and family violence (DFV) in our society? In addition, what may be its forms of presentation in health facilities?
2. To address these types of violence related issues, what are the activities being conducted currently? For effective management of this issue, what do you think need to be done? What health care providers or health facilities need to do?
3. Do you think, the intervention we have provided had contributed to improve the self-confidence, manage common problems and stress and use the support services or resources at times of need? If yes, in what way has it helped?
4. In your opinion, what are the good and strong points of this program? How such type of programs contribute to the wellbeing of abused women, or to health facilities and in total, general public health?
5. What do you think are the weaknesses of this program or the aspects to be improved?
6. How can we integrate this program to regular health service being provided by the hospital?
   1. How can the existing physical infrastructure and process contribute to the continuity of this program?
   2. In order to give continuity to this program, what additional things we need? What are your suggestions and recommendations?
7. What might be the barriers or difficulties that can arise while participating in this programme?
   1. In regards to patients: safety issues related to their participation, confidentiality
   2. In regards to hospital: shortage of staff, physical infrastructure, money, time management, networking with support organisations
8. How health facilities and health organisations can contribute to make this program sustainable and effective?
9. Based on the policies and regulations of this hospital, what are the provisions for initiating a new program here? (Only for managerial level respondents)
