# Supplementary material for: ‘We don’t see because we don’t ask’: Qualitative exploration of service users’ and health professionals’ views regarding a psychosocial intervention targeting pregnant women experiencing domestic and family violence
Source: PLoS One. 2020 Mar 9;15(3):e0230069. doi: 10.1371/journal.pone.0230069 (PMC7062263; doi:10.1371/journal.pone.0230069)
Supplement: Attachment — Submitted filename: Responses_reviewers_R2_14022020.docx [file pone.0230069.s004.docx]

**Response to reviewers’ comments**

The authors would like to thank the editor and reviewers for their careful and thorough reading of this manuscript and for their thoughtful comments and constructive feedback. Please find responses to the comments below. Changes to the manuscript are presented in track changes format within the revised document.

| **S.N.** | **Reviewers’ comments** | **Responses** |
| --- | --- | --- |
| A. | **Reviewer 2:** Thank you for addressing issues raised in the previous review. Upon re-reviewing the manuscript, however, I would like to raise issues that I feel still require further response. | |
| 1. | There appears to be inconsistency in the stated aims of the research.  The abstract (lines 25-26) - "acceptability and the perceived impact of....[the intervention]  Finding - Line 382 - 'improvement and sustainability'. Although this is not an aim per se, it does highlight the inconsistency across the manuscript in what was being addressed through this particular study. Clarity and consistency needed.  The introduction (lines 135-119) - "....perceived strengths and limitations of [the intervention] (described as 'objectives' | Thank you. We appreciate the reviewer’s comments.  To assess acceptability of any intervention, it is necessary to identify the perceived impact of that intervention (strengths) and barriers in implementing that intervention. In addition, exploring recommendations and suggestion from participants will help to design the intervention in the most effective way for future implementation. Hence, in the abstract section, general objective was stated and it was further broken down into specific objectives. (Page no. 7, line no. 115-119)  This study has two specific objectives.   - To explore the perceived strengths and limitations of the counselling and psychoeducation intervention as experienced by intervention participants and HCPs. - To solicit feedback on what elements of the intervention and process could be improved and how.   The objective in the abstract was restructured to make it clearer and consistent. (Page no. 2, line no. 26-28)  As suggested, the sentence was restructured to ensure consistency. (Page no. 19, line no. 388-89) |
| 2. | Line 133, reference is made to "the research nurse", yet line 139 refers to "Both interviewers'. It is unclear how many interviewers there were (1 or 2) and whether the 'research nurse' is one of them. Clarity is needed. | Thank you for the information.  The research nurse (RP) had interviewed participants from the intervention group and the intervention nurse (DS) had interviewed HCPs.  This had been mentioned in the Data Collection section and to make it clearer, initials of both interviewers have been added in the text.  (Page no. 8, line no. 134, 137, 141) |
| 3. | Lines 277 and 282 - the term 'participants' is used. 'Participants' refer to BOTH the HCPs and women. If the discussion is about how the women felt, then I would anticipate that 'participant' should actually be women. A similar issue arises throughout the theme "Impact of intervention on women's lives" | Thank you for your feedback.  This study had explored perspectives of both the service providers and service users. It is not just about how women felt but also how service providers felt about the intervention. Hence, ‘participants’ term was used to indicate both of them and either woman or HCP was used after each quote to make the reader better understand which quote was said by whom. |
| 4. | Line 169 - what is meant by "reasoned, logical and valid" | Thank you for pointing this out.  The interpretation made in the result section was rigorously discussed among the research team to ensure it is logical and valid. Furthermore, appropriate reasoning was put forward to support the findings presented.  As suggested the sentence was restructured to make it clearer. (Page no. 9; line no. 171) |
| 5. | Lines 179-180 : it is noted that "A review of field notes in conjunction with the transcripts addressed dependability". Dependability is normally addressed through the keeping of an audit trail. It is unclear how merely reviewing notes and transcripts addresses dependability. Explanation (with references) needed. | Thank you for pointing this out.  This section has been amended. (Page no. 10; line no. 182-84) |
| 6. | Line 274 - 'Verbal feedback' is referred to. Should this not be data from the interviews?  What is meant by 'fair proportion' -line 344 | Thank you for your feedback. This has been revised as suggested. (Page no. 14; line no. 279)  The proportion is now quantified to make it clearer. (Page no. 17; line no. 348) |
| 7. | There is still some disconnect between the surrounding text and the quotes provided as evidence:  Eg  Lines 224 - 225. The main thrust of the quote is family relations but the text refers to lack of education | Thank you for your feedback. In this section, some contributing factors of DFV as expressed by participants were presented. The sentence was restructured to make it clearer. (Page no. 12; line no. 226-227)  Other quotes were also reviewed and arranged appropriately to ensure that the surrounding text align with the quotes provided. |
| 8. | Lines 287 - 288. This quote is about 'confidence'. The link to how a person centred approach enables women to come forward is still unclear. | Thank you for your feedback.  Person-centered approach would encourage women to express themselves without hesitation and address their individual needs and concerns. This approach would improve confidence among women to share their problem with HCPs. |
| 9. | Lines 289 - 291 - the quote which refers to 'contained space' (I'm assuming is quite removed from where this was discussed in the text. Please consider inserting. | Thank you for your feedback. There is a quote referring to contained space in Page no. 15; line no. 294-296. |
| 10. | Lines 353 - 354 - Why is there a quote from a woman when the preceding text is related to HCPs? | Thank you for your feedback. Explanatory text was added before the quote to make it clearer. (Page no. 18; line no. 358-59) |
| 11. | Lines 429 - 434 - the preceding text refers to collaboration with stakeholders, supportive supervision and debriefing are discussed but different issues are raised in the quotes | Thank you for your feedback.  ‘All higher authorities need to be involved’ (Page no. 21; line no. 440) – This implies collaboration with the stakeholders at all level for proper implementation of the program. |
| 12. | Line 463 - how does 'adequate time' related to 'the best quality care' as described in the preceding text. | When there will be a decrease in patient load, health care providers will get adequate time for discussing with patients, which is crucial for improving the effective doctor-patient relationship. This approach would ensure correct identification and management of the patient’s issue. However, failure to allocate adequate time for patient’s consultation might raise several issues such as misdiagnosis, miscommunication and lack of empathy from the doctor leading to distrust from the patients.  (Ref: <https://praxhub.com/are-your-patients-receiving-adequate-time-and-quality-care/>) |
| 13. | Written English. Although it is noted that the manuscript has been copy edited, there are a number of instances throughout where further work is required. |  |
|  | Eg:  Abstract:  Lines 36-37 : "Intervention participants expressed the counselling session as a safe haven....." Awkward expression | Thank you for your feedback.  The sentence has been restructured to make it clearer. (Page no. 2; line no. 37-38) |
|  | Line 64 - consider not using the term 'cases' to refer to women' (ie, the first time 'cases' is mentioned in the sentence) | Thank you for your feedback. The suggested change has been made. (Page no. 4; line no. 62) |
|  | Line 70 - The term 'programmes insufficient' is used. Are the programs insufficient or the number (or spread) of programs insufficient? See also line 83 | Thank you for your feedback. The sentence has been restructured to make it clearer.  (Page no. 4; line no. 70-72; Page no. 5; line no 83-84) |
|  | Line 72 - what is meant by the 'enforcement' of the initiatives. Please consider using a more relevant term | Thank you for your feedback. The sentence has been restructured to make it clearer.  (Page no. 4; line no. 72-73) |
|  | Line 74 - Please reconsider the phrase 'larger segment of population in Nepal' - Awkward expression | Thank you for your feedback. The sentence has been restructured to make it clearer. (Page no. 5; line no. 74-75) |
|  | Line 79 - I a review 'on' intervention, or 'about' interventions | Thank you. Change has been made as suggested. (Page no. 5; line no. 79) |
|  | Line 85 - What is meant by 'alert women against DFV' - Awkward expression. | Thank you for your feedback. The sentence has been restructured to make it clearer. (Page no. 5; line no. 85) |
|  | Line 90 - we screen victims 'ford' DFV not 'of DFV' | Thank you. Change has been made as suggested. (Page no. 5; line no. 90) |
|  | Line 96 - change 'involvements' to 'involvement' | Thank you. Change has been made as suggested. (Page no. 5; line no. 96) |
|  | Line 151 - "....were read in conjunction with the transcribed verbatim". It appears word/s are missing. | Thank you for your feedback. The sentence has been restructured to make it clearer. (Page no. 8-9; line no. 152-54) |
|  | Line 179 - What is meant by "a triangulation of researchers"? | Triangulation of researchers means involvement of multiple researchers/observers with different areas of expertise. This approach enables securing as many different views as possible during the analysis of the qualitative data (Denzin, 1978). Available in <https://qualpage.com/2018/01/18/triangulation-in-qualitative-research/> |
|  | Line 213 - Do you mean,.....'and overlapping in nature'? | Thank you. It has now been corrected. (Page no. 11; line no. 217) |
|  | Line 256 - change 'currently' to at the time the article was written, as this information may go out of date. | Thank you. It has now been corrected. (Page no. 13; line no. 258-59) |
|  | Line 259 - insert 'the' between 'notify' and 'hospital's' | Thank you. Change has been made as suggested. (Page no. 13; line no. 263) |
|  | Line 301-302 - ...learn how to respond to FV and some topics | Thank you. We appreciate the reviewer’s comments. We have rechecked the line highlighted to ensure it is correct. |
|  | Line 387 0 delete 'as well' | Thank you. As suggested, ‘as well’ was deleted. (Page no. 19; line no. 394) |
|  | Line 515 - insert 'the' between 'of' and 'booklet' | Thank you. As suggested, ‘the’ was added. (Page no. 25; line no. 522) |
|  | Line 516- change 'after' to 'rather' | Thank you. Change has been made as suggested. (Page no. 25; line no. 524) |
|  | Line 518 - insert 'a' between 'DFV' and 'one-size-fits-all" | Thank you. Change has been made as suggested. (Page no. 25; line no. 526) |
|  | Line 520 - change 'influences' to 'influence' | Thank you. Change has been made as suggested. (Page no. 25; line no. 528) |
|  | Line 544- meaning of 'awareness on need of acting against DFV' is unclear | Thank you for your feedback. The sentence has been restructured to make it clearer. (Page no. 26; line no. 552-54) |
|  | Line 545 - delete 'very' | Thank you. The necessary amendment was made. (Page no. 26; line no. 553) |
|  | Line 552 - this sentence reads as if the HCPs are disclosing the women's experiences. I'm sure this is not what was intended | Thank you. The sentence has now been restructured to make it clearer. (Page no. 27; line no. 559-62) |
|  | Line 559 - delete 'the' | Thank you. The necessary amendment was made. (Page no. 27; line no. 568) |
|  | Line 564 - do you mean 'funding in this area'? | Thank you. The necessary amendment was made. (Page no. 27; line no. 573) |
|  | Line 573, delete the comma after 'Despite' | Thank you. As suggested, a comma was added. (Page no. 28; line no. 582) |
|  | Line 581. Start a new sentence at 'this study' | Thank you for your feedback (Page no. 28; line no. 594) |
| **B.** | **Reviewer’s 4 comments** |  |
|  | This qualitative study examines the acceptability and utility of a psychosocial intervention, from the perspective of service users and health professionals. Overall, the authors have addressed the reviewers’ prior concerns and comments. | |
| 1. | I’m not sure why the authors have chosen to use DFV for Domestic & Family Violence since the more common terms (and therefore more searchable by potential readers) are DV (domestic violence) or IPV (intimate partner violence). I recommend the authors use the most commonly used IPV. | Thank you. We appreciate the reviewer’s suggestion. We do agree that IPV is the most common term, however, all of the authors have had discussions about the best terminology to be used in the article at the time of the protocol development. Agreement on using DFV was based on the understanding that in the setting where the study was conducted, (i.e. Nepal, the perpetrators include not only the husband, but other family members, including father, mother, and brother-in-laws. This has been described in the protocol paper which has been published elsewhere. The link to the protocol paper is  <https://bmjopen.bmj.com/content/9/4/e027436> |
| 2. | While significantly improved over the initial submission, the manuscript requires copyediting for language usage, grammar and clarity. Related to this, some of the quotes were poorly translated into English. | The manuscript has been thoroughly reviewed and grammatical errors have been rectified. Two of the authors are native English speakers who have reviewed again for English edits. |
